# Plasma metabolites associated with homeostatic model assessment of insulin resistance: metabolite-model design and external validation

**DOI:** 10.1038/s41598-019-50260-7

**Published:** 2019-09-25

**Authors:** Pablo Hernández-Alonso, Jesús García-Gavilán, Lucía Camacho-Barcia, Anders Sjödin, Thea T. Hansen, Jo Harrold, Jordi Salas-Salvadó, Jason C. G. Halford, Silvia Canudas, Mònica Bulló

**Affiliations:** 1 Human Nutrition Unit, Faculty of Medicine and Health Sciences, Institut d’Investigació Sanitària Pere Virgili, University Hospital Sant Joan de Reus, Rovira i Virgili University, Reus, Spain; 2 CIBER Fisiopatología de la Obesidad y Nutrición (CIBEROBN), Instituto de Salud Carlos III, Madrid, Spain; 3 Department of Nutrition, Exercise and Sports, University of Copenhagen, Copenhagen, Denmark; 4 Department of Psychological Sciences, Institute of Psychology Health and Society, University of Liverpool, Liverpool, UK

**Keywords:** Metabolomics, Type 2 diabetes

## Abstract

Different plasma metabolites have been related to insulin resistance (IR). However, there is a lack of metabolite models predicting IR with external validation. The aim of this study is to identify a multi-metabolite model associated to the homeostatic model assessment (HOMA)-IR values. We performed a cross-sectional metabolomics analysis of samples collected from overweight and obese subjects from two independent studies. The training step was performed in 236 subjects from the SATIN study and validated in 102 subjects from the GLYNDIET study. Plasma metabolomics profile was analyzed using three different approaches: GC/quadrupole-TOF, LC/quadrupole-TOF, and nuclear magnetic resonance (NMR). Associations between metabolites and HOMA-IR were assessed using elastic net regression analysis with a leave-one-out cross validation (CV) and 100 CV runs. HOMA-IR was analyzed both as linear and categorical (median or lower versus higher than the median). Receiver operating characteristic curves were constructed based on metabolites’ weighted models. A set of 30 metabolites discriminating extremes of HOMA-IR were consistently selected. These metabolites comprised some amino acids, lipid species and different organic acids. The area under the curve (AUC) for the discrimination between HOMA-IR extreme categories was 0.82 (95% CI: 0.74–0.90), based on the multi-metabolite model weighted with the regression coefficients of metabolites in the validation dataset. We identified a set of metabolites discriminating between extremes of HOMA-IR and able to predict HOMA-IR with high accuracy.

## Introduction

Insulin resistance (IR) can lead to a number of increased health risks, including type 2 diabetes (T2D), cardiovascular disease (CVD) and other metabolic conditions^1^. There are different approaches to estimate IR. The hyperinsulinemic-euglycemic clamp (HEC) is the current gold-standard test for assessing IR once it is established but it is time-consuming, expensive, and rarely used outside of a clinical research setting^2^. An alternative to HEC is the homeostatic model assessment of IR (HOMA-IR) based on a spot fasting plasma glucose and insulin^3^, which values significantly correlate (r = 0.88) with those obtained with HEC^3^. However, no simple fasting blood test currently exists for the prediction of IR before its onset.

To this regard, specific circulating metabolites are postulated as early predictors of an impaired IR status^2^. Association of branched-chain amino acids (BCAAs) and aromatic amino acids with obesity and IR was first reported more than 40 years ago by Felig *et al*.^4^. However, new evidences suggest that metabolites, either altered by or predicting IR, do not belong to a unique group of molecules. In fact, apart from amino acids (AA; e.g. BCAAs, glutamic acid (Glu), glycine (Gly), serine (Ser), threonine (Thr) and cysteine (Cys)), different lipid species (phosphatidylcholines (PC), sphingomyelins (SM)), specific fatty acids (FA; i.e. linoleic acid, stearic acid, palmitic acid) and other organic acids (2- and 3-2-hydroxybutyrate) can be considered as candidate biomarkers for discriminating IR status^2,5^. Despite this, there are no studies predicting IR through a multi-metabolite model.

The aim of this study is to report a set of metabolites discriminating impaired IR status. Moreover, we aimed to validate the multi-metabolite model in an external population with similar characteristics and to investigate whether this multi-metabolite model may predict baseline and/or changes in HOMA-IR with high accuracy in two independent populations.

## Results

### Characteristics of the studies

Table 1 shows the general characteristics of the SATIN and GLYNDIET participants. Median value for HOMA-IR was 1.84 and 1.04 in the SATIN and GLYNDIET cohorts, respectively. As shown, similar demographic, body composition and biochemical data were found between both study populations. The two datasets differed in subjects’ age, body weight, glucose levels, insulin levels and HOMA-IR, whereas no significant differences according to sex distribution, BMI, WC and the lipid profile were shown (Table 1). Supplementary Fig. 1 shows the flowchart of both study populations. A total of 236 (SATIN) and 102 (GLYNDIET) subjects were included in the analysis.

**Table 1:** Baseline characteristics of the SATIN and GLYNDIET studies.

Variable	SATIN	GLYNDIET	*P*-value
Sample size, N	236	102	—
Age, years	46.37 ± 10.65	44.01 ± 7.75	0.025
Female sex, % (N)	78.81 (186)	80.39 (82)	0.855
Body weight (kg)	87.48 ± 11.16	83.06 ± 10.05	<0.001
Body mass index (kg/m^2^)	31.1 ± 2.15	30.97 ± 2.15	0.605
Waist circumference (cm)	101.01 ± 9.4	100.73 ± 7.69	0.773
Glucose (mg/dL)	93.25 ± 11.03	101.57 ± 14.67	<0.001
Insulin (mIU/L)	10.25 ± 8.88	5.08 ± 3.04	<0.001
HOMA-IR	2.43 ± 2.22	1.34 ± 1.11	<0.001
Median HOMA-IR^*^	1.84 [1.18–3.17]	1.04 [0.80–1.75]	—
Total cholesterol (mg/dL)	196.01 ± 34.88	193.05 ± 30.97	0.439
HDL-C (mg/dL)	55.65 ± 15.27	54.89 ± 11.13	0.609
LDL-C (mg/dL)	119.88 ± 30.51	117.25 ± 28.09	0.442
Triglycerides (mg/dL)	102.34 ± 48.9	100.83 ± 59.75	0.822

Mean ± SD, unless otherwise stated. Abbreviations: *median [IQR]; -C, cholesterol; HDL, high-density lipoprotein; HOMA-IR, homeostasis model assessment of insulin resistance; LDL, low-density lipoprotein.

### Selection of metabolites

**Figure 1:**
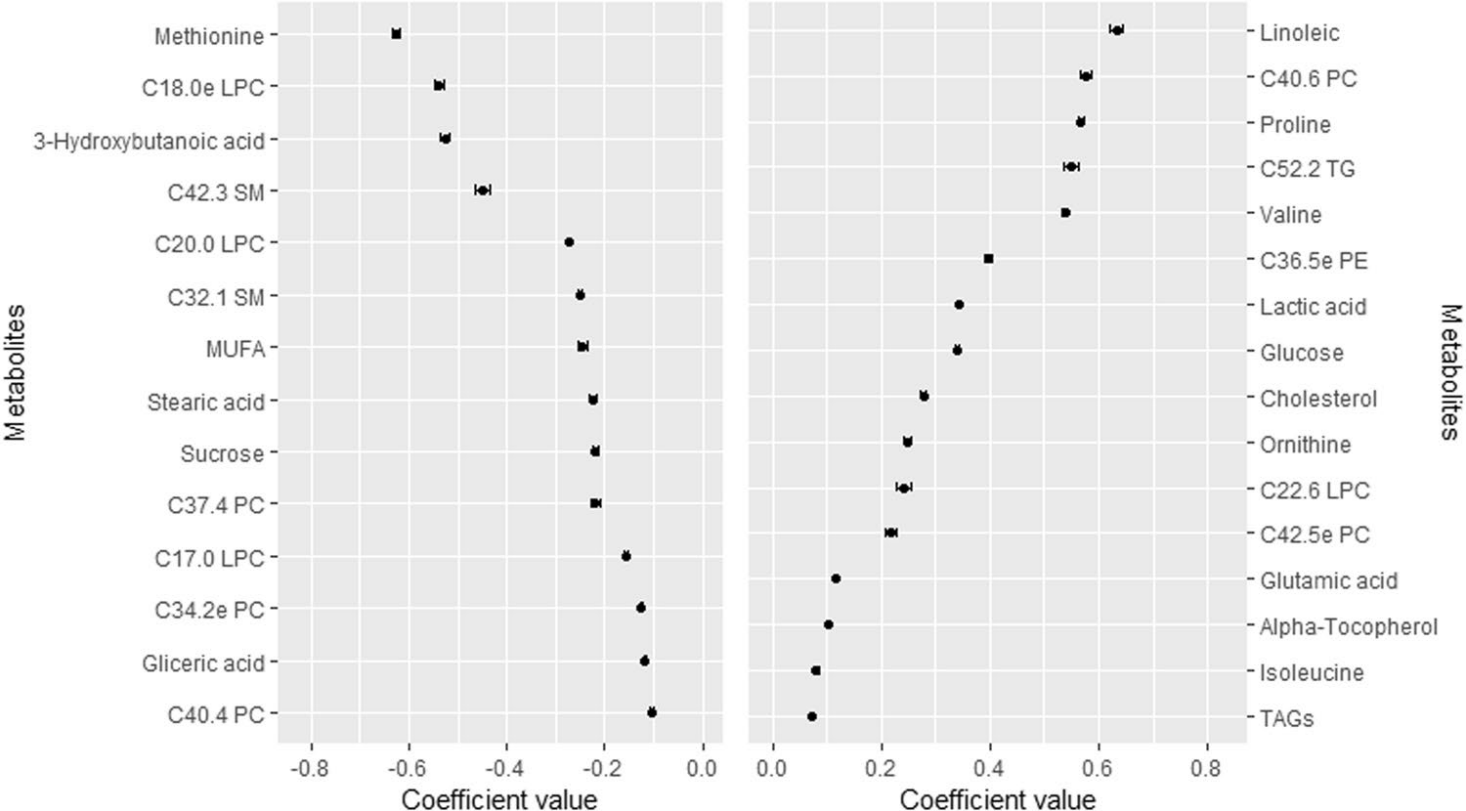
Metabolites consistently selected in the 100 times iteration of the elastic net logistic regression using the whole SATIN dataset. Data shows median and 95% CI in the 100 iterations from the elastic net logistic regression. Abbreviations: e, ether-linked isobaric species of plasmanyl analogue of glycerophospholipids; LPC, lysophosphatidylcholine; PC, phosphatidylcholine; PE, phosphatidylethanolamine; SM, sphingomyelin; TG and TAGs, triglycerides.

**Figure 2:**
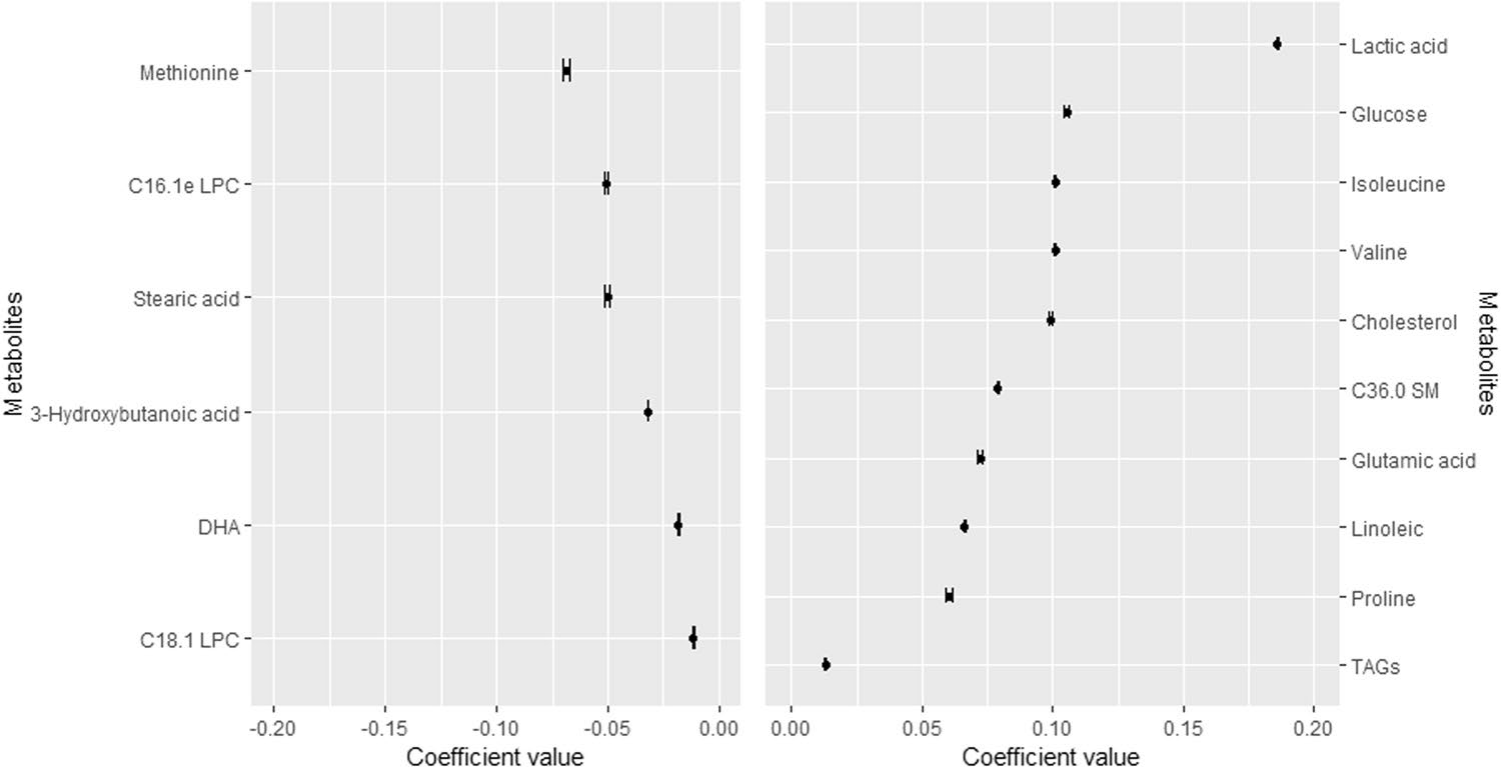
Metabolites consistently selected in the 100 times iteration of the elastic net Gaussian regression using the whole SATIN dataset. Data shows median and 95% CI in the 100 iterations from the elastic net Gaussian regression. Abbreviations: DHA, docosahexaenoic acid; e, ether-linked isobaric species of plasmanyl analogue of glycerophospholipids; LPC, lysophosphatidylcholine; SM, sphingomyelin; TAGs, triglycerides.

A total of 30 metabolites were consistently selected in the 100 iterations in the elastic net logistic regression using the SATIN dataset (Fig. 1). Methionine (Met), C18:0e LPC, 3-hydroxybutanoic acid and C42:3 SM were those with the highest negative coefficients, whereas linoleic acid, C40:6 PC and proline (Pro) were those with the highest positive coefficients. When the elastic net Gaussian regression was used, 16 metabolites were consistently selected (Fig. 2). Supplementary Table 1 shows the full list of metabolites selected at least 1 time in the elastic net logistic and Gaussian regression together with the median and 95% CI of each coefficient in each model. Notably, alanine, C38:5 and C36:1 PCs and fructose were selected 95 times out of 100 in case of the binary approach and C36:5e PE was selected 97 times out of 100 in case of continuous regression approach. Metabolites selected using both approaches mostly comprised amino acids and lipid species such as SMs, lysophosphatidylcholines (LPC), PCs, TGs and phosphatidylethanolamines (PE). Importantly, consistency between both approaches was found for those metabolites positively or negatively associated with HOMA-IR.

### Predictability of the models

**Figure 3:**
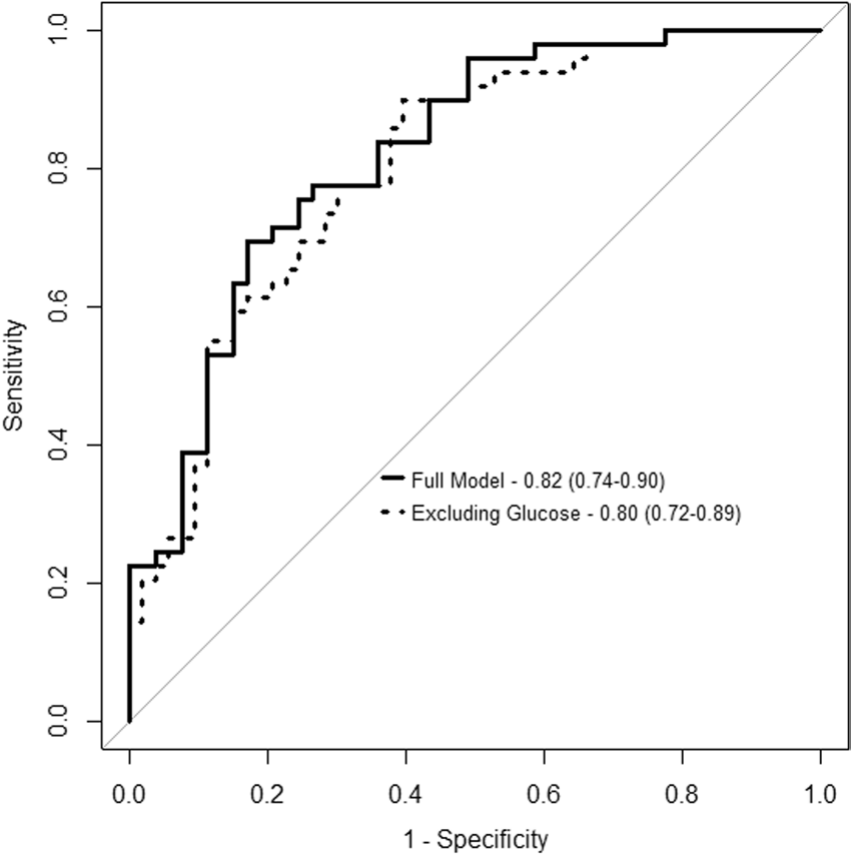
Area under the curve (AUC) in the validation dataset (GLYNDIET study).

We assessed the ability of the multi-metabolite model to predict HOMA-IR as a categorical variable. The AUC (95% confidence interval (CI)) was 0.85 (0.80–0.90) in the intra-SATIN-validation dataset using the LOO-CV approach. This multi-metabolite model predicted HOMA-IR in the external validation GLYNDIET dataset with an AUC (95% CI) of 0.82 (0.74–0.90) (Fig. 3). A similar and non-significantly different predictive ability was found after excluding glucose - before running the elastic net logistic regression - from the model with an AUC (95% CI): 0.86 (0.82–0.91 in the intra-SATIN validation dataset and 0.80 (0.72–0.89) in the GLYNDIET dataset.

We additionally analyzed the predictive ability using HOMA-IR as a continuous variable. The model derived from the elastic net Gaussian regression was assessed in the intra-SATIN-validation dataset using the LOO-CV approach. It showed a Pearson coefficient (r) of 0.43 (95% CI: 0.32–0.53), and an adjusted R^2^ of 0.308. By applying this model in the external validation GLYNDIET dataset, we obtained coefficients of r = 0.51 (95% CI: 0.35–0.64) and R^2^ = 0.378, respectively. After excluding glucose from the model, results for SATIN were r = 0.38 (95% CI: 0.26–0.48) and R^2^ = 0.24, whereas in the GLYNDIET r = 0.40 (95% CI: 0.23–0.56) and R^2^ = 0.290.

### Analysis of changes in HOMA-IR

**Figure 4:**
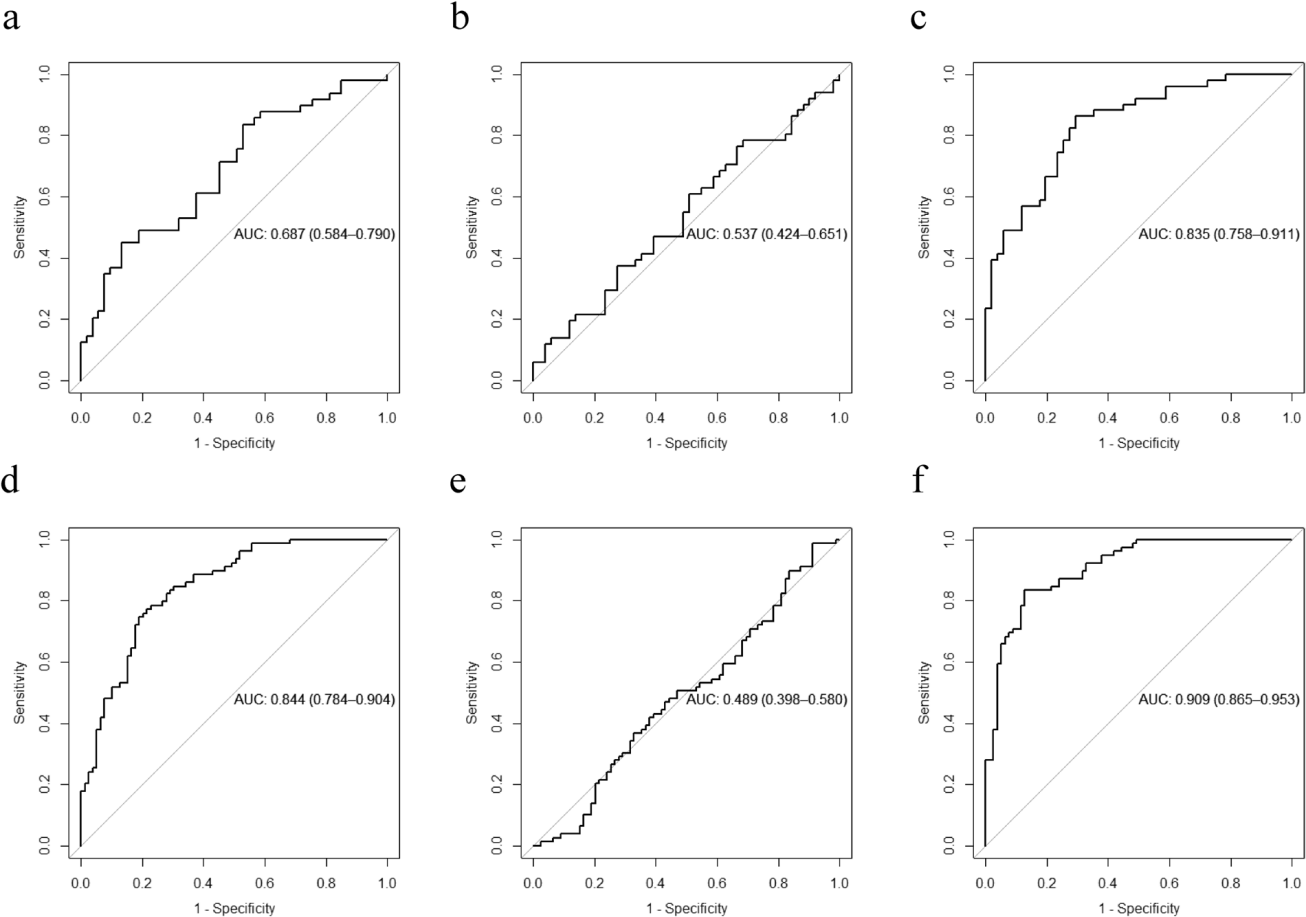
Area under the curve (AUC) in the longitudinal analysis. AUC was computed in the whole datasets using median as the cut-off point in the GLYNDIET (**a**–**c**) and SATIN (**d**–**f**) datasets. Sections refer to: raw HOMA-IR changes (**a**,**d**); HOMA-IR changes adjusted by changes in BW (**b**,**e**); and HOMA-IR changes adjusted by changes in BW and baseline HOMA-IR (**c**,**f**). BW, body weight; HOMA-IR, homeostasis model assessment of insulin resistance.

We additionally used the multi-metabolite model built in the SATIN dataset (Fig. 1) to explore whether it might be useful to predict changes in HOMA-IR in both the GLYNDIET (6 months length) and SATIN (2 months length) studies. We evaluated the predictive ability of i) non-adjusted HOMA-IR changes, ii) HOMA-IR changes adjusted by changes in body weight and iii) HOMA-IR changes adjusted by changes in body weight and baseline HOMA-IR. These models were tested according to the changes in HOMA-IR median categories (Fig. 4). In both studies the AUCs had the highest value in the full adjusted model (Fig. 4c,f), thus indicating that the multi-metabolite model was affected by the initial HOMA-IR values and that when those initial HOMA-IR values are known, the multi-metabolite model can accurately predict an increase or decrease in HOMA-IR values.

## Discussion

In the present analysis, we have identified a quantitative multi-metabolite model consisting of specific amino acids, lipid species and other organic acids that discriminates extremes of HOMA-IR with a high accuracy in two independent study cohorts. This multi-metabolite model has also a good ability to predict changes in HOMA-IR.

To identify early biomarkers of IR could be a good strategy to manage this metabolic dysregulation thus preventing diseases^6^. Previous quantitative metabolomic studies have shown that different plasma metabolites including lipid species and AAs are altered in the IR status^7–11^. However, others failed to find a different metabolite profile between subjects with and without IR^12^. A recent study identified several plasma metabolites predicting changes in insulin sensitivity in obese, nondiabetic subjects from the Diet, Obesity, and Genes (DiOGenes) Study^10^. They identified a 27-metabolite panel discriminating glycemic improvers (i.e. Matsuda index improvements ≥40.36%) with an AUC (95% CI) of 0.77 (0.70–0.85). A simplification of the previous 27-metabolite model (Pro and C34:1 PC) plus Matsuda Index showed an AUC of 0.75 (0.67–0.83). More recently, in the Korean Genome and Epidemiology study^11^ a specific serum metabolite profile was associated with incident T2D. Similar to these previous studies, we also described a significant association between several AAs and lipids with HOMA-IR, and our multi-metabolite model showed a marginal and non-significant higher predictability (AUC of 0.82 (0.74–0.90)) than those in the previous studies. In our study, glucose, TAGs, Pro, C36:5e PE, C32:1, C36:1 and C42:5 PCs displayed a positive association with HOMA-IR, whereas Met and C40:4 PC were negatively associated. However, most of the lipid species from the aforementioned studies were not found in our CV analysis maybe due to collinearity with other species selected by the model.

In our analysis, both cholesterol and TGs levels were positively associated with HOMA-IR. Although mechanisms linking glucose and lipid metabolism are not well-known, low absorption and high synthesis of cholesterol are associated with high circulating glucose levels, IR, and obesity^13^. In fact, upregulated cholesterol synthesis has been associated with peripheral IR independent of obesity^14^ and fasting TGs are useful clinical predictor of IR and T2D development^15^. Not only cholesterol and TGs but also other lipids could be associated to IR. A LC-MS lipid profiling conducted in 189 T2D incident cases and their 1:1 matched controls in the Framingham Heart Study after 12 years of follow-up showed a lipid pattern for T2D risk^16^. They showed that C52:1 TG was linked with an increased risk of T2D, whereas C38:6 PC was linked with a decreased risk. According to these results, we found C52:2 TG as positively associated to HOMA-IR, whereas C38:5 PC as negatively associated. Interestingly, a set of highly unsaturated lipid species were positively associated with HOMA-IR, whereas a set of saturated or slightly unsaturated lipid species shown a negative association with HOMA-IR. The relevance of the length and degree of unsaturation of the FAs belonging to the different lipid species deserves a deeper investigation to unravel their interplay and role in IR status.

In contrast to published findings, linoleic acid was found positively and stearic acid negatively associated with HOMA-IR. Low linoleic acid concentrations have been positively associated with HOMA-IR^17,18^. In fact, those subjects in the highest quintile of insulin-sensitive showed a significantly higher percentage of linoleic acid^19^. However, some controversy exists as muscle membrane linoleic acid has also been positively related to IR^20^. Moreover, despite supplementation with conjugated linoleic acid (CLA) had positive effects on HDL metabolism, it has been described to have adverse effects on insulin and glucose metabolism. It increases fasting glucose concentrations and HOMA-IR^21^. In a cross-sectional study, researchers explored the association of certain red blood cells FAs with different parameters of insulin metabolism in 625 subjects. Stearic acid was associated with fasting glucose but not associated with fasting insulin or HOMA-IR^22^. Therefore, even though SFA have been previously associated with IR and glucose intolerance (i.e. risk factor for T2D), a more complex homeostasis is possibly taking place. In fact, a recent study showed that a low fat diet (LFD) led to an increase in very-LDL (VLDL) palmitic (C16:0), stearic (C18:0), and palmitoleic (C16:1n7c) acids, while no changes were observed on the HFD, usually linked to induced IR.

Several studies have suggested that IR plays a key role in the link between metabolic disorders and increased circulating concentrations of BCAAs^23,24^. Insulin is a regulator of branched-chain α-keto acid dehydrogenase (BCKDH) complex^25^, a rate-limiting enzyme of BCAA catabolism. Therefore, a suppression of BCAA catabolism by IR is considered as a plausible etiology of elevated BCAA concentration in obesity. According to two previous studies conducted in healthy^7^ and T2D^8^ Japanese subjects, several AAs such as alanine (Ala), isoleucine (Ile), Glu, Pro and valine (Val) displayed a positive association with HOMA-IR in our study. However, contrary to the previous findings, we additionally reported a negative association between Met and HOMA-IR. Organic acids including lactate and glyceric acid have been linked to IR in accordance with previous findings. Blood lactate was found elevated among obese, IR and T2D subjects and decreased following weight loss^26,27^. One prospective study further suggested that serum lactate may be an independent risk factor for the development of T2D^28^. In contrast, glyceric acid is inversely associated to insulin levels and showed a trend in case of HOMA-IR^29^.

In our analysis, 3-hydroxybutanoic acid (aka beta-hydroxybutyrate (βOHB)) – hepatic ketone body – displayed a negative association with HOMA-IR. Hydroxycarboxylic acid receptor 2 (HCAR2) binds and is activated by βOHB [55], which diminishes the rate of lipolysis in adipocytes^30^. For βOHB, this might denote a feedback mechanism to regulate accessibility of the FA precursors of ketone body metabolism. However, elevated plasma free FAs from adipocytes with metabolic impairments are thought to affect IR by diverse mechanisms related to inflammation and oxidative stress^31^. Thus, activation of HCAR2 by βOHB might has a role in the improvement of glucose control and in the amelioration of some macrovascular complications of T2D.

The positive association we found between alpha-tocopherol and HOMA-IR deserves some comments. Observational studies suggest that reduced plasma vitamin E levels are associated with an increased risk of developing T2D^32,33^ and IR^34^. However, although a transient IR improvement in overweight subjects supplemented with vitamin E for 3 months has been reported^35^, its effects at long term and/or in T2D subjects are still inconclusive^36^.

Our study has some strengths and limitations that deserve further attention. First, the study was conducted in participants with overweight or obesity, thus our findings may not be comprehensive to participants with differing weight status. Second, we did not examine all the lipids, thus we cannot discard different modulations by other means. Third, we were not able to discriminate between specific isomers in some lipid species, nor between the FA content of some lipids such as PCs and LPCs. Importantly, HOMA-IR can be considered an “inaccurate” method for assessing IR. However, individuals from extreme categories of HOMA-IR had significant differences in TGs and HDL-C levels supporting clear differences in insulin sensitivity between categories. Among the strengths are the use of two independent cohort studies to design and validate our metabolite models. Moreover, the use of a multi-platform and targeted methodology allowed the coverage of a broad plasma metabolomics range. The use of internal and external standards made possible the determination of their plasma concentration.

In conclusion, the present study shows that a specific 30 multi-metabolite model discriminates between subjects with higher and lower HOMA-IR and has the ability to predict changes in HOMA-IR in two independent study populations. Despite the limited number of existing multi-metabolite models for HOMA-IR prediction, our results mirror specific metabolites or certain type of metabolites previously reported. However, future studies should extend this approach by establishing a small set of metabolites that could be clinically assessed and lead to a practical metabolomics-based HOMA-IR tool.

## Methods

Two independent study populations were included in the present analysis. The SATIN study is a multicentre 2-month weight-loss trial and it was used as a training set for the analysis. The GLYNDIET study is a 6-month weight-loss trial that was used as a validation set for the model obtained in the training set.

### Study subjects

Participants from the SATIN study were community-dwelling adults recruited in Spain and Denmark from 2015 to 2016. They were aged 20–65 years (mean ± SD: 46.37 ± 10.65), with a body mass index (BMI) 27.0–35.0 kg/m^2^ (31.1 ± 2.15), fat mass ≥23% and without associated comorbidities. The study was registered in clinicaltrials.gov as NCT02485743. Participants in the GLYNDIET study were community-dwelling adults, aged 30–60 years (44.01 ± 7.75) with a BMI ranging from 27.0–35.0 kg/m^2^ (30.97 ± 2.15) who were recruited in Spain from 2010 to 2012. GLYNDIET trial was registered in www.controlled-trials.com as ISRCTN54971867. Exclusion criteria in both clinical trials included recent changes in body weight (±3 kg in the last three months), chronic kidney and liver pathologies, diseases which may affect energy expenditure, current or former (<3 months) smoking, and regular consumption of alcohol above the recommendations. All procedures were conducted in accordance with the ethical principles set forth in the current version of the Declaration of Helsinki (Fortaleza, Brazil, October 2013) and the International Conference on Harmonization E6 Good Clinical Practice (ICH-GCP). The protocols were approved by the local Institutional Review Boards and Ethics Committees of all the recruiting centres (Denmark: De Videnskabsetiske Komiteer for Region Hovedstaden; Spain: Committee for Ethical Clinical Investigation (CEIC), Hospital Universitari Sant Joan de Reus) and all participants provided written informed consent.

### Data collection and anthropometric, nutritional and biochemical measurements

Anthropometric measures (BMI, waist circumference (WC)) were taken by trained personnel with calibrated equipment. Assessment of dietary intake was based on three-day dietary record (3-DDR) which included two workdays and a weekend day. These were inspected for clarification immediately upon receipt by trained dietitians. Energy and nutrient intake were estimated using national site specific food composition Tables^37–39^. Plasma fasting glucose and insulin concentrations were measured using standard enzymatic automated methods and HOMA-IR was computed^3^. Plasma fasting serum total cholesterol, high-density lipoprotein (HDL) cholesterol, low-density lipoprotein (LDL) cholesterol and triglyceride (TG) concentrations were determined by using standard enzymatic automated methods (COBAS; Roche Diagnostics Ltd). In subjects whose TG concentrations were <400 mg/dL, LDL-cholesterol concentration was estimated by using Friedewald’s formula.

### Metabolomic procedures: multiplatform targeted metabolomics

The same metabolomics procedures and platforms were used in both studies. These have been previously published^40^. Metabolomic analysis was conducted in frozen samples from the two studies at the same time by using a common protocol. Plasma metabolite profiling included an automated metabolite extraction and a multiplatform analysis using three different methodologies: Proton nuclear magnetic resonance (^1^H NMR), liquid chromatography coupled to mass spectrometry (LC-MS) and gas chromatography coupled to mass spectrometry (GC-MS). To normalize the signaling from different samples throughout the entire analysis, internal standards were used for LC-MS and GC-MS. We used Electronic Reference To access *In vivo* Concentrations 2 (ERETIC) based on Pulse Length–based Concentration determination (PULCON) using an external 2 mM sacarose standard in order to calibrate NMR signal and quantify the metabolites^41^.

### Automation of multiple plasma sample extraction

Aqueous extractions of 250 µL of plasma were performed with a methanol/water solution in a Bravo automated liquid handling platform (Agilent Technologies, CA). Lipid extractions of 100 µL of plasma were performed by a biphasic extraction with methanol/methyl-tert-butyl ether (MTBE). Solvents were added automatically to the samples and after the appropriate shaking and centrifugation steps the supernatants were dispensed in 96-well plates and stored until analysis with GC-MS, LC-MS/MS and NMR. The lipidic extraction protocol has some different procedure for samples to NMR or LC-MS determinations. For NMR measurement, serum lipidic extracts were dried and then were reconstituted in 0.01% TMS (tetramethylsilane) solution (0.067 mM) of 2:1 CD_3_Cl (chloromethane-d3):CD_3_OD (methanol-d4) with a 4% of D_2_O (deuterium oxide) (EreticSignal 6.166 mM). For LC-MS measurement, serum lipidic extracts were diluted with methanol. For GC-MS analysis, sample extracts were previously two-step derivatized by methoximation and silylation. Internal standards for GC and LC were previously dispensed to the same plates where supernatants were collected. Quality controls (i.e. pool of samples) were used in both GC and LC to discard drift in the instrumental response.

### Lipid ^1^H-NMR profiling

Samples were prepared and extracted and the regions identified (following the procedure described in Vinaixa *et al*.^42^) and compared directly with lipid standards. ^1^H-NMR spectra were recorded at 300 K on an Avance III 600 spectrometer (Bruker, Germany) operating at a proton frequency of 600.20 MHz using a 5 mm PBBO gradient probe. Lipid samples were measured and recorded in PROCNO 11 using a simple pre-saturation sequence (recycle delay (RD)-90°-ACQ pre-saturation pulse (zgpr) program) to eliminate the residual water moisture of deuterated methanol. After pre-processing and visual checking of the NMR dataset, specific ^1^H regions of diacylgycerols, TGs and total lipids based on terminal methyl and methylene signals were identified in the spectra using a comparison in the AMIX 3.9 software (Bruker, Germany). Curated identified regions across the spectra were integrated using the same AMIX 3.9 software package and exported to excel spreadsheets to obtain relative concentrations.

### Lipid LC-MS profiling

The lipid species in plasma samples were determined by ultra-high performance liquid chromatography (UHPLC) coupled to quadrupole-time of flight (qTOF) high-resolution MS (6550 iFunnel series, Agilent Technologies, Spain). The ionization was performed in positive electrospray, and the mass calibration reference was used in all the analyses to keep the mass accuracy below 5 ppm. Lipids were separated in a C18 reversed phase column (Kinetex C18-EVO from Phenomenex) and a ternary mobile phase (water/methanol/2-propanol) was used. Each lipid was quantified with an internal standard calibration method using one analytical standard and one deuterated internal standard for each lipid family.

### Aqueous GC-MS profiling

Samples were analysed in a 7890 A Series GC coupled to a triple quadrupole (QqQ) (7000 series; Agilent Technologies, Barcelona, Spain). The chromatographic column was a J&W Scientific HP5-MS (30 m × 0.25 mm i.d., 0.25 µm film; Agilent Technologies, Barcelona, Spain), and helium (99.999% purity) was used as a carrier gas. Ionization was carried out with electronic impact recording data in “Full Scan” mode.

Quantification was performed by internal standard calibration, using the corresponding analytical standard for each determined metabolite and a deuterated internal standard depending on the family of metabolite. The internal standards used were succinic d_4_ acid, glycerol ^13^C_3_, norvaline, L-methionine-(carboxy-^13^C,methyl-d_3_), D-glucose ^13^C_6_, myristic-d_27_ acid and alpha-tocopherol d_6_.

### Statistical analyses

Baseline characteristics of study participants are described as means and standard deviations (SD) or median (95% confidence interval (CI)) for quantitative variables, and percentages (numbers) for categorical variables. Comparison of baseline characteristics between studies were performed using Welch’s t-test to account for the unequal variances and sample sizes. Values for metabolites with less than 20% missing values were imputed using the random forest imputation approach (“missForest” v.1.4^43^ R package), otherwise they were not included in the analysis (m = 10). The levels of 123 metabolites were first centered and scaled using the standard deviation as the scaling factor^44^.

Subjects were categorized based on median HOMA-IR values and a dichotomous variable was created according to median values or lower versus higher than the median values. The multi-metabolite model was selected in the SATIN study and the external validation was conducted on the GLYNDIET study using its own HOMA-IR median value. Importantly, an internal validation was also performed in the SATIN dataset.

Due to the collinear nature of the data and the number of metabolites exceeding the number of observations, logistic regression with elastic net penalty (implemented in the “glmnet” v.2.0-16^45^ R package) was used to build a discrimination model for HOMA-IR binary variable. A 100-fold cross-validation (CV) was performed to find the optimal value of the tuning parameter that resulted in the minimum mean-squared error (MSE)^45^. The value minMSE was estimated using the argument s = “lambda.min” in the cv.glmnet function. The discrimination model scores were computed as the weighted sum of all metabolites with weights equal to the regression coefficients from the discrimination models.

We additionally used the leave-one out (LOO) CV approach to obtain the unbiased estimates of these model scores and avoid overfitting when the model was internally validated in the SATIN population. Briefly, in each run, the elastic net method was applied to N-1 of the samples (training sets) and the model obtained was applied to the remaining excluded sample (intra-validation sets) in case of the SATIN dataset.

The area under curve (AUC, implemented in the “pROC” v.1.13.0^46^ and “cvAUC” v.1.1.0^47^ R packages) was computed to assess the accuracy of the predicted models in the validation sets.

We additionally performed linear regression analysis with elastic net penalty (i.e. elastic Gaussian regression). Prior autoscaling to correct variable variance, we used HOMA-IR as continuous variable to examine the association with metabolites. The variance of HOMA-IR explained by the metabolites was estimated from the adjusted R^2^ by including all selected metabolites in the model. We also computed the Pearson correlation coefficients between the reported and predicted (using elastic net Gaussian regression) HOMA-IR levels. All analyses were performed using R statistical software v3.4.0^48^.

## Supplementary information

Supplementary material
