# Supplementary material for: Plasma metabolites associated with homeostatic model assessment of insulin resistance: metabolite-model design and external validation
Source: Sci Rep. 2019 Sep 25;9:13895. doi: 10.1038/s41598-019-50260-7 (PMC6761105; doi:10.1038/s41598-019-50260-7)
Supplement: Supplementary file 1 — Supplementary material [file 41598_2019_50260_MOESM1_ESM.docx]

**SUPPLEMENTARY INFORMATION**

***Plasma metabolites associated with homeostatic model assessment of insulin resistance: metabolite-model design and external validation***

**Authors:** Pablo Hernández-Alonso, Jesús García-Gavilán, Lucía Camacho-Barcia, Anders Sjödin, Thea T. Hansen, Jo Harrold, Jordi Salas-Salvadó, Jason C. G. Halford, Silvia Canudas and Mònica Bulló.

**Supplementary Table 1.** List of metabolites selected at least one time in the 100 cross-validation using the whole SATIN dataset.

|  | Elastic logistic regression (HOMA-IR using median division) | | Elastic Gaussian regression (Linear HOMA-IR) | |
| --- | --- | --- | --- | --- |
| **Metabolites** | **# times out of 100** | **Median (95% CI)** | **# times out of 100** | **Median (95% CI)** |
| Methionine | 100 | -0.626 (-0.634; -0.618) | 100 | -0.068 (-0.07; -0.067) |
| 3-Hydroxybutanoic acid | 100 | -0.527 (-0.535; -0.518) | 100 | -0.032 (-0.032; -0.032) |
| Stearic acid | 100 | -0.224 (-0.231; -0.218) | 100 | -0.05 (-0.051; -0.049) |
| TAGs | 100 | 0.073 (0.072; 0.074) | 100 | 0.013 (0.013; 0.013) |
| Isoleucine | 100 | 0.08 (0.076; 0.085) | 100 | 0.101 (0.101; 0.101) |
| Glutamic acid | 100 | 0.117 (0.115; 0.119) | 100 | 0.072 (0.071; 0.073) |
| Cholesterol | 100 | 0.278 (0.274; 0.283) | 100 | 0.099 (0.098; 0.1) |
| Glucose | 100 | 0.341 (0.337; 0.344) | 100 | 0.105 (0.104; 0.106) |
| Lactic acid | 100 | 0.343 (0.342; 0.343) | 100 | 0.186 (0.186; 0.186) |
| Valine | 100 | 0.538 (0.531; 0.544) | 100 | 0.101 (0.1; 0.101) |
| Proline | 100 | 0.567 (0.562; 0.571) | 100 | 0.06 (0.059; 0.061) |
| Linoleic | 100 | 0.632 (0.619; 0.645) | 100 | 0.066 (0.066; 0.067) |
| C36.5e PE | 100 | 0.397 (0.39; 0.404) | 97 | 0.006 (0.005; 0.006) |
| Gliceric acid | 100 | -0.119 (-0.121; -0.116) | 79 | -0.001 (-0.001; -0.001) |
| Sucrose | 100 | -0.22 (-0.226; -0.214) | 21 | 0.003 (0.002; 0.003) |
| C18.0e LPC | 100 | -0.539 (-0.549; -0.528) | 0 | NA (NA; NA) |
| C42.3 SM | 100 | -0.448 (-0.463; -0.433) | 0 | NA (NA; NA) |
| C20.0 LPC | 100 | -0.273 (-0.279; -0.268) | 0 | NA (NA; NA) |
| C32.1 SM | 100 | -0.25 (-0.253; -0.247) | 0 | NA (NA; NA) |
| MUFA | 100 | -0.246 (-0.255; -0.237) | 0 | NA (NA; NA) |
| C37.4 PC | 100 | -0.22 (-0.229; -0.21) | 0 | NA (NA; NA) |
| C17.0 LPC | 100 | -0.158 (-0.162; -0.155) | 0 | NA (NA; NA) |
| C34.2e PC | 100 | -0.126 (-0.129; -0.124) | 0 | NA (NA; NA) |
| C40.4 PC | 100 | -0.104 (-0.108; -0.1) | 0 | NA (NA; NA) |
| Alpha-Tocopherol | 100 | 0.104 (0.103; 0.105) | 0 | NA (NA; NA) |
| C42.5e PC | 100 | 0.219 (0.209; 0.229) | 0 | NA (NA; NA) |
| C22.6 LPC | 100 | 0.241 (0.228; 0.255) | 0 | NA (NA; NA) |
| Ornithine | 100 | 0.247 (0.24; 0.254) | 0 | NA (NA; NA) |
| C52.2 TG | 100 | 0.55 (0.536; 0.563) | 0 | NA (NA; NA) |
| C40.6 PC | 100 | 0.576 (0.567; 0.585) | 0 | NA (NA; NA) |
| Alanine | 95 | 0.012 (0.011; 0.013) | 1 | 0.004 (NA; NA) |
| C38.5 PC | 95 | -0.08 (-0.086; -0.074) | 0 | NA (NA; NA) |
| C36.1 PC | 95 | 0.043 (0.04; 0.046) | 0 | NA (NA; NA) |
| Fructose | 95 | 0.067 (0.062; 0.072) | 0 | NA (NA; NA) |
| C33.1 PC | 86 | 0.055 (0.046; 0.065) | 0 | NA (NA; NA) |
| C32.1e PC | 66 | 0.023 (0.019; 0.027) | 0 | NA (NA; NA) |
| C16.1 LPC | 37 | -0.04 (-0.047; -0.033) | 0 | NA (NA; NA) |
| Citric acid | 37 | 0.015 (0.011; 0.02) | 0 | NA (NA; NA) |
| C16.0 LPC | 7 | -0.01 (-0.024; 0.003) | 21 | 0 (-0.002; 0.001) |
| Glycolic acid | 7 | -0.01 (-0.02; -0.001) | 0 | NA (NA; NA) |
| Glycerol | 7 | -0.004 (-0.023; 0.015) | 0 | NA (NA; NA) |
| LPC | 7 | 0.015 (-0.006; 0.036) | 0 | NA (NA; NA) |
| Tryptophan | 2 | -0.027 (-0.066; 0.011) | 0 | NA (NA; NA) |
| C54.2 TG | 1 | -0.022 (NA; NA) | 0 | NA (NA; NA) |
| Free Cholesterol | 1 | -0.014 (NA; NA) | 0 | NA (NA; NA) |
| Phenylalanine | 1 | -0.012 (NA; NA) | 0 | NA (NA; NA) |
| C40.5e PC | 1 | 0.017 (NA; NA) | 0 | NA (NA; NA) |
| C38.1 SM | 1 | 0.029 (NA; NA) | 0 | NA (NA; NA) |
| C16.1e LPC | 0 | NA (NA; NA) | 100 | -0.051 (-0.051; -0.05) |
| C18.1 LPC | 0 | NA (NA; NA) | 100 | -0.012 (-0.012; -0.011) |
| C36.0 SM | 0 | NA (NA; NA) | 100 | 0.079 (0.079; 0.079) |
| DHA | 0 | NA (NA; NA) | 100 | -0.018 (-0.019; -0.018) |
| C38.5e PC | 0 | NA (NA; NA) | 83 | 0.007 (0.006; 0.007) |
| C38.2 SM | 0 | NA (NA; NA) | 1 | -0.006 (NA; NA) |

**Abbreviations**: DHA, docosahexaenoic acid; e, ether-linked isobaric species of plasmanyl analogue of glycerophospholipids; LPC, lysophosphatidylcholine; MUFA, monounsaturated fatty acid; NA, not available; PC, phosphatidylcholine; PE, phosphatidylethanolamine; SM, sphingomyelin; TG and TAG, triglyceride.

**Supplementary Figure 1.** Flowchart of the study populations


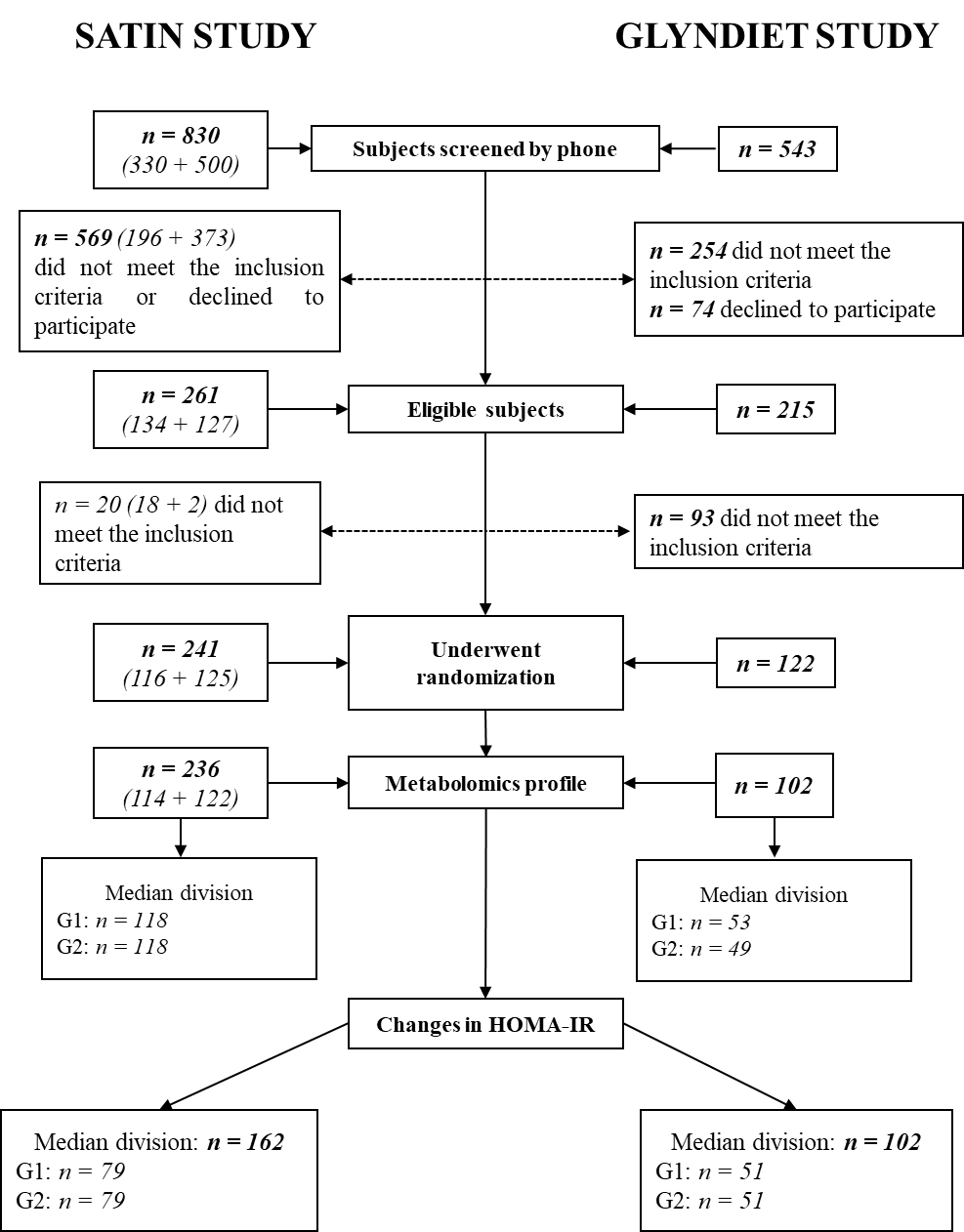


In the SATIN study, numbers in parenthesis represent data from the Danish and Spanish nodes, respectively. G1 (median value or lower) and G2 (higher than the median value).
